# Functional Analyses of Two Novel *LRRK2* Pathogenic Variants in Familial Parkinson′s Disease

**DOI:** 10.1002/mds.29124

**Published:** 2022-06-16

**Authors:** Ilda Coku, Eugénie Mutez, Sabiha Eddarkaoui, Sébastien Carrier, Antoine Marchand, Claire Deldycke, Liesel Goveas, Guillaume Baille, Mélissa Tir, Romain Magnez, Xavier Thuru, Gaëlle Vermeersch, Wim Vandenberghe, Luc Buée, Luc Defebvre, Bernard Sablonnière, Marie‐Christine Chartier‐Harlin, Jean‐Marc Taymans, Vincent Huin

**Affiliations:** ^1^ University of Lille, Inserm, CHU Lille, U1172—LilNCog (JPARC)—Lille Neuroscience & Cognition Lille France; ^2^ University of Lille, Inserm, CHU Lille, Expert Center for Parkinson's Disease Lille France; ^3^ Department of Neurology and Expert Center for Parkinson's Disease Amiens University Hospital, CHU Amiens‐Picardie Amiens France; ^4^ University of Lille, CNRS, Inserm, CHU Lille, UMR9020‐U1277—CANTHER—Cancer Heterogeneity Plasticity and Resistance to Therapies Lille France; ^5^ Department of Neurology AZ Sint‐Lucas Bruges Belgium; ^6^ Department of Neurology University Hospitals Leuven Leuven Belgium; ^7^ Laboratory for Parkinson Research, Department of Neurosciences KU Leuven Leuven Belgium; ^8^ University of Lille, Inserm, CHU Lille, Department of Toxicology and Genopathies UF Neurobiology Lille France

**Keywords:** Parkinson's disease, *LRRK2*, mutation, kinase, genetics

## Abstract

**Background:**

Pathogenic variants in the *LRRK2* gene are a common monogenic cause of Parkinson's disease. However, only seven variants have been confirmed to be pathogenic.

**Objectives:**

We identified two novel *LRRK2* variants (H230R and A1440P) and performed functional testing.

**Methods:**

We transiently expressed wild‐type, the two new variants, or two known pathogenic mutants (G2019S and R1441G) in HEK‐293 T cells, with or without LRRK2 kinase inhibitor treatment. We characterized the phosphorylation and kinase activity of the mutants by western blotting. Thermal shift assays were performed to determine the folding and stability of the LRRK2 proteins.

**Results:**

The two variants were found in two large families and segregate with the disease. They display altered LRRK2 phosphorylation and kinase activity.

**Conclusions:**

We identified two novel *LRRK2* variants which segregate with the disease. The results of functional testing lead us to propose these two variants as novel causative mutations for familial Parkinson's disease. © 2022 The Authors. *Movement Disorders* published by Wiley Periodicals LLC on behalf of International Parkinson and Movement Disorder Society

## 1 Introduction

Parkinson's disease (PD) is a neurodegenerative disorder characterized by the selective loss of dopaminergic neurons from the substantia nigra pars compacta associated with Lewy bodies rich in aggregated alpha‐synuclein and lipids in surviving neurons.^1^ Most cases are sporadic. However PD can be concentrated in certain families and/or have an early‐onset (≤45 years). It can be caused by a monogenic form of the disease explaining <10% of familial cases and a still lower frequency of apparently sporadic cases.^2^, ^3^, ^4^

**FIG 1:**
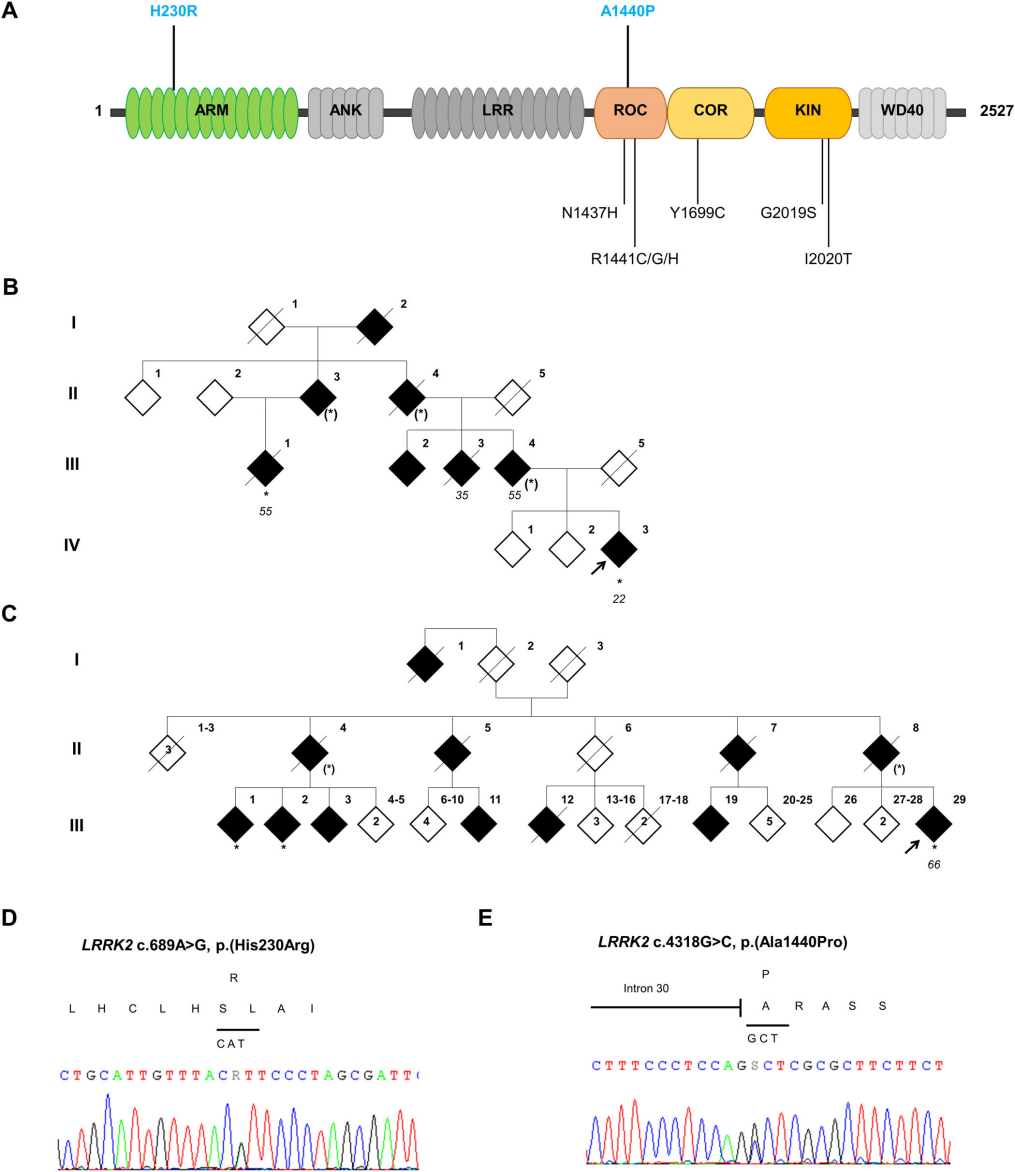
Nature and position of two novel *LRRK2* pathogenic variants. (A) Schematic linear representation of LRRK2 protein. The two novel pathogenic variants are indicated in bold above the protein and the seven known pathogenic mutants are indicated below the protein. Each domain of LRRK2 is named: ARM, armadillo repeats; ANK, ankyrin repeats; LRR, leucine‐rich repeats; ROC, Ras of complex proteins GTPase; COR, C‐terminal of ROC; KIN, kinase; WD40, WD40 repeats. **(B)** Family tree of family 1, with the *LRRK2* H230R variant. (**C)** Family tree of family 2, with the *LRRK2* A1440P variant. The probands are denoted by a black arrow. Filled black symbols denote clinically affected members and open symbols indicate unaffected individuals. / = deceased, * = genotyped carrier, (*) = obligatory carrier. The numbers under each individual correspond to the age of onset of PD. Family pedigrees have been anonymized for confidentiality. (**D, E**) Electropherograms of heterozygous pathogenic variants of *LRRK2* for NM_198578.3:c.689A>G, p.(His230Arg) **(D)** and NM_198578.3:c.4318G>C, p.(Ala1440Pro) **(E)**. [Color figure can be viewed at wileyonlinelibrary.com]

Pathogenic variants in the leucine‐rich repeat kinase 2 (*LRRK2*) gene are among the most common genetic causes of familial and sporadic PD. Indeed, the G2019S pathogenic variant is the most frequent, with its prevalence reaching up to 29% in Ashkenazi Jewish and 37% in North African Berber populations.^5^ More than 80 rare coding sequence variants in *LRRK2* have been reported to be linked to PD thus far,^6^ but only seven (ie, N1437H, R1441G, R1441C, R1441H, Y1699C, G2019S, and I2020T) (Fig. **1**A) have been confirmed to be pathogenic and responsible for PD with a Mendelian inheritance.^5^ To date, all pathogenic variants are located in the kinase or Roc‐COR domains.

The major hypothesis to explain the pathophysiology of *LRRK2* pathogenic variants in PD is a gain of function that induces an increase in kinase activity and hyperphosphorylation of the substrate proteins.^7^, ^8^ Altered autophosphorylation of serine 1292^9^ and more significantly the increased Rab proteins phosphorylation have been observed in *LRRK2* pathogenic variants in cellulo and are indicators of kinase activity.^8^ Other biomarkers of LRRK2 activity are the phosphorylation by other upstream kinases (i.e., hetero‐phosphorylation) of a cluster of phosphorylation sites located between the ANK and LRR domains, including serines 935 and 910. These phosphorylations are reported to affect LRRK2 properties, such as LRRK2 complex formation, subcellular localization, and binding with 14–3‐3 protein,^10^, ^11^ but do not correlate with LRRK2 kinase activity.

Here, we identified two novel *LRRK2* variants: H230R in the armadillo domain and A1440P in the ROC domain. We tested the kinase activity of these new variants in cellulo and assessed their thermal stability to demonstrate their pathogenicity.

## 2 Patients and Methods

### 2.1 Subjects

Two families displaying PD with an autosomal‐dominant inheritance pattern were screened during targeted next‐generation sequencing of PD genes in a diagnostic setting (Fig. 1B,C). We enrolled and sampled five affected patients (Fig. 1B). Patients underwent a detailed clinical evaluation in the department of Neurology and Expert Center for Parkinson's disease at the Lille, Amiens, Bruges, or Leuven Hospitals. Clinical diagnoses were reviewed according to the international diagnostic criteria for PD.^12^ Extensive genetic analyses were performed to eliminate other genetic diseases (Supplementary material). All individuals gave their written informed consent. The study was conducted according to the French ethics regulations (Lille Ethics Committee, Protocole Convergence, CPP/2008/009).

### 2.2 Functional Testing

Briefly, we used a previously described plasmid construct^13^, ^14^ for the wild‐type (WT) human LRRK2 (pLV‐CSJ‐3FLAG‐LRRK2‐WT) as a template to introduce the two novel variants (H230R and A1440P) and two known pathogenic variants (G2019S and R1441G) as positive controls. We transiently expressed WT, or the LRRK2 mutants, in HEK‐293 T (human embryonic kidney cells that express the SV40 large T antigen) cells, with and without LRRK2 kinase inhibitor.^15^ We performed western blot to asses LRRK2 hetero‐ and auto‐phosphorylation, and to characterize the phosphorylation of a known LRRK2 substrate, RAB10 at threonine 73. Lastly, we purified the LRRK2 proteins and performed thermal shift assay.

Additional methods are described in the Supplementary material.

## 3 Results

### 3.1 Genetic Analyses

Next‐generation sequencing revealed two missense variants in *LRRK2* (NM_198578.3): c.689A>G, p.(His230Arg) in exon 6 in family 1 (Fig. 1B,D) and c.4318G>C, p.(Ala1440Pro) in exon 31 in family 2 (Fig. 1C,E). There were no pathogenic variants in the other PD genes. These variants were absent from databases of healthy individuals (gnomAD v3.1.1).^16^ The variant A1440P is located in a mutational hotspot in the Roc domain and multiple prediction tools (DANN, MutationTaster, FATHMM, GERP++, LRT, MetaLR, MetaSVM, PROVEAN, SIFT, Polyphen 2, MutationAssessor, and the Rare Exome Variant Ensemble Learner score) favored a deleterious effect. Segregation analyses showed the variant A1440P to be heterozygous in two affected cousins of the proband and the variant H230R to be heterozygous in the proband's second cousin affected with PD. Mutation prediction tools provided contradictory results. Co‐segregation analyses in these two families provided a moderate level of evidence of pathogenicity.^17^ Details of clinical phenotypes and results from mutation prediction tools are provided in Supplementary data.

### 3.2 Kinase Activity

**FIG 2:**
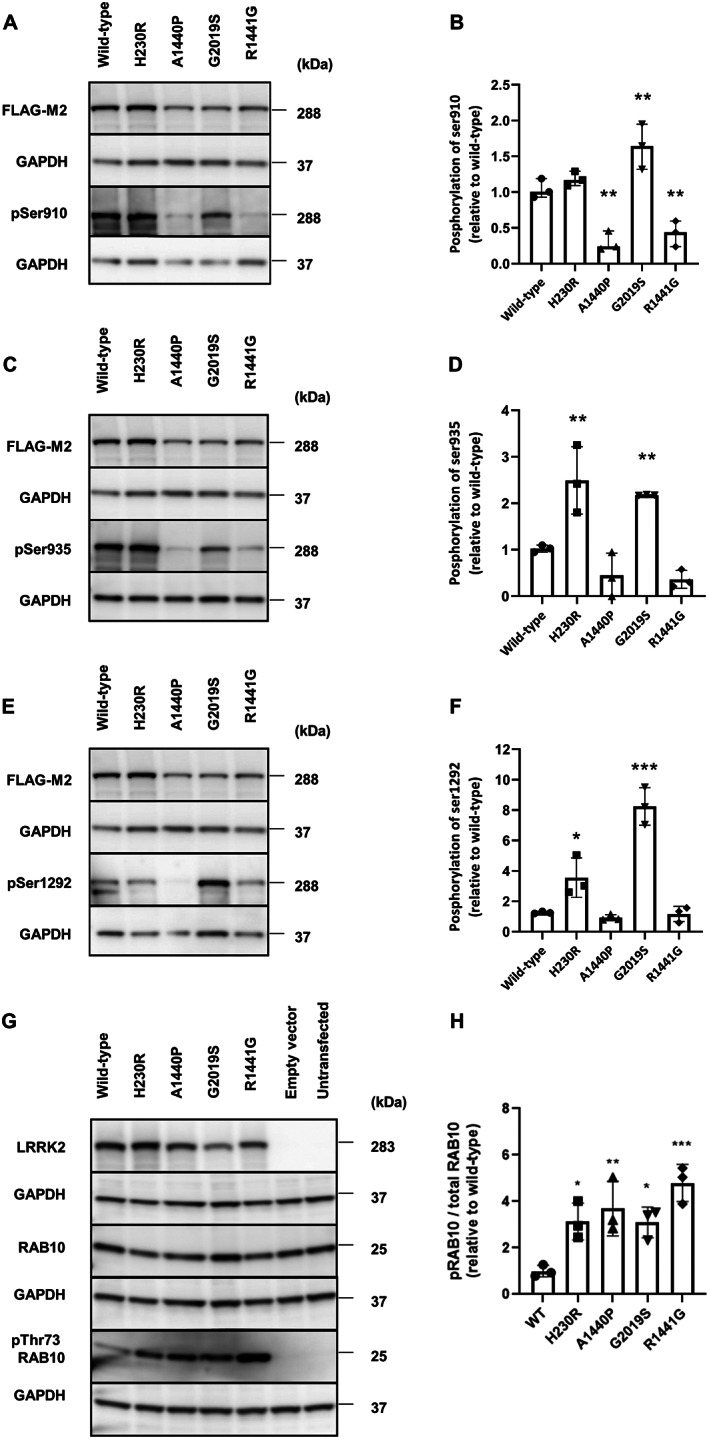
Comparison of phosphorylation sites in Parkinson's disease ( **PD)** ‐associated mutants. Representative western blots and quantification of LRRK2 phosphorylation at serines 910 **(A, B)**, 935 **(C, D)**, and 1292 **(E, F)** in WT and LRRK2 mutants. Representative western blot **(G)** and quantification of RAB10 phosphorylation at threonine 73 **(H)** for the WT and LRRK2 mutants. Error bars indicate the standard deviation of replicates (n = 3). kDa = kilodalton. *P < 0.05, **P < 0.01, ***P < 0.001; ****P < 00001.

We first studied the phosphorylation of LRRK2 by other kinases at serine 910 and 935 (Fig. 2A–D). In HEK‐293 T cells transiently expressing WT or mutant forms of LRRK2, WB analyses showed a higher phosphorylation rate for G2019S than for WT at serine 910 (1.6‐fold increase, *P* = 0.005) and serine 935 (2.1‐fold increase, *P* = 0.016), whereas the R1441G mutant showed 2.5‐fold lower phosphorylation at serine 910 (*P* = 0.009), as expected,^11^, ^18^ and a nonsignificant decrease in phosphorylation at serine 935 (*P* = 0.215). The H230R mutant showed a slight but nonsignificant increase in phosphorylation at serine 910 (*P* = 0.561) and a 2.4‐fold increase at serine 935 (*P* = 0.003), whereas the mutant A1440P showed 3.5‐fold decreased phosphorylation at serine 910 (*P* = 0.003) and a slight but nonsignificant decrease in phosphorylation at serine 935 (*P* = 0.319).

We then studied the phosphorylation of serine 1292 (Fig. 2E‐F), which is an indicator of LRRK2 autophosphorylation and kinase activity. As expected, we observed higher phosphorylation of serine 1292 for the mutant G2019S (6.6‐fold increase, *P* < 0.0001).^9^ R1441G showed no difference relative to WT (*P* = 0.997). The mutant H230R showed 2.8‐fold greater phosphorylation at serine 1292 (*P* = 0.013), whereas A1440P showed no difference relative to WT (*P* = 0.999).

We next characterized the phosphorylation of a known LRRK2 substrate, RAB10 at threonine 73. We do not observe in HEK‐293 T cells a significant endogenous expression of LRRK2. Moreover, endogenous Rab10 is not phosphorylated at Thr73 in the absence of LRRK2 in our cellular model (Fig. 2G, Fig. S1). We observed 3.1‐fold higher phosphorylation for G2019S (*P* = 0.029) and 4.8‐fold higher phosphorylation for R1441G (*P* < 0.0006). The rate of phosphorylation of RAB10 was approximately 3.1‐fold higher for H230R and 3.7‐fold higher for A1440P (*P* < 0.026 and *P* = 0.007, respectively) (Fig. 2G,H).

### 3.3 LRRK2 Inhibitor

Finally, we investigated the kinase activity of our mutants using MLi‐2, a highly selective LRRK2 kinase inhibitor, and observed how the inhibition of LRRK2 protein kinase affects the phosphorylation of LRRK2 or its substrate RAB10. We compared HEK‐293T cells transiently expressing WT or mutant forms of LRRK2 and treated with 100 nM MLi‐2 or 0.01% DMSO for 1 h (Fig. S2). Under all conditions, the cells treated with MLi‐2 showed less mean phosphorylation than those treated with DMSO. There was significantly less phosphorylation of serine 910 for G2019S (*P* = 0.013), serine 935 for H230R (*P* = 0.003) and G2019S (*P* = 0.032), and serine 1292 for H230R (*P* = 0.042) and G2019S (*P* = 0.006). Treatment with MLi‐2 almost completely suppressed the phosphorylation of RAB10 at threonine 73 under all conditions (Fig. S3).

## 4 Discussion

We identified two novel potentially disease‐causing variants of the *LRRK2* gene from large autosomal‐dominant PD families. Unfortunately, we were unable to test the presence of the two new variants in all affected and unaffected family members. The genotypes of the parents and grandparents were only assumed. Therefore, the segregation analyses were limited despite the size of the families. Our segregation analyses combined with the characteristics of the variants (frequency in databases, location in the gene, prediction tools) provide sufficient evidence to consider them to be at least “likely pathogenic” for A1440P according to the American College of Medical Genetics (ACMG) guidelines for the classification of genetic variants.^19^ Addition of functional analyses, segregation analyses, prediction tools, and the frequency of these variants in databases of healthy individuals reinforce the arguments in favor of a pathogenic effect.

*LRRK2* A1440P shows phosphorylation rates at serine 910, 935, and 1292 similar to those of R1441G located at the adjacent position.^9^, ^11^ Conversely, H230R shows a pattern of phosphorylation more similar to that of G2019S.^9^, ^11^ All previously reported pathogenic LRRK2 mutants show greater phosphorylation of Rab proteins.^20^, ^21^ Similarly, we observed approximately two‐fold greater phosphorylation of RAB10 at threonine 73 for both mutants than for WT LRRK2. These results are comparable with those of previous studies, suggesting that LRRK2 kinase activity cannot be uniformly predicted by its autophosphorylation and cellular phosphorylation site status.^22^

Limitations of our study are the use of a transient gene expression and the method of detection we used. Our study should be confirmed in other models as inducible stable expression system and/or using other quantification methods such as quantitative multiplexed immunoblot using fluorescently‐labeled secondary antibodies or measurement of LRRK2‐dependent Rab10 phosphorylation in patient‐derived peripheral blood neutrophils by immunoblotting as well as by targeted mass spectrometry.^23^

Given its location and the similar phosphorylation pattern of the mutated protein, the pathological effects of A1440P are likely to be similar to those of pathogenic variants located in the Roc‐COR domain, such as R1441G. However, the pathophysiology and increased kinase activity of the H230R variant, located in the ARM domain, is less obvious. Another reported variant in this domain, A211V, also showed a slight increase in kinase activity.^24^, ^25^ Kishore et al. identified A397T, G472R, and L550W variants but they did not describe the kinase activity of these rare variants.^26^ Another rare variant, N551K, belonging to a protective haplotype (N551K‐R1398H‐K1423K) has been reported for PD patients^27^, ^28^ but the mechanisms explaining how this haplotype confers neuroprotection in PD is not clear and it has not been functionally assessed. Only one transcriptomic study in *Drosophila melanogaster* has identified altered pathways associated with N551K, including alterations of the oxidoreductase pathway.^29^ Structural analysis of full‐length human LRRK2 has shown that the ANK and LRR domains interact with the kinase domain but not the ARM domain, which shows flexibility relative to the rest of the protein.^30^ Rab proteins directly interact with LRRK2 via the ARM domain^31^, ^32^ but the H230R variant is not located in the potential Rab‐interacting regions of this domain (residues 386–392).^30^ It has also been suggested that amino acid substitutions of the conserved ARM domain of LRRK2 enhance interactions with FADD and induce apoptosis via caspase‐8.^33^ Another study reported that LRRK2 interacts with Hsp90 via its ARM domain and then Hsp90 subsequently interacts with the E3 ubiquitin ligase CHIP to decrease LRRK2 CHIP‐mediated degradation.^34^ The ARM domain interacts with RAB7L1 (RAB29),^32^ a membrane‐anchored RAB GTPase that recruits LRRK2 to the trans‐Golgi network or lysosomes via the ANK domain and highly stimulates its kinase activity.^32^

In conclusion, we have identified two novel *LRRK2* variants, H230R and A1440P, which segregate with the disease in large PD families. We show that H230R and A1440P alter the phosphorylation rates of LRRK2 and its ability to phosphorylate its substrate RAB10. Further studies on these rare potentially disease‐causing variants should help us to better understand how LRRK2 dysfunction causes PD and may have implications for future treatment strategies against *LRRK2*‐related disorders.

## Conflict of Interest

The authors declare that there is no conflict of interest.

## Funding Information

This work was funded by the University of Lille, the “Institut National de la Santé et de la Recherche Médicale” (INSERM), the Lille University Hospital, the Association des Aidants et Malades à Corps de Lewy (A2MCL) charity, and the France Alzheimer charity. This work was also supported by grants from the Programs d'Investissements d'Avenir LabEx (excellence laboratory) DISTALZ (Development of Innovative Strategies for a Transdisciplinary approach to ALZheimer's disease), the ANR (Agence Nationale de Recherche, France) grant ANR‐16‐CE16‐0012‐02 MeTDePaDi, grant ANR‐20‐CE16‐0008 Synapark, grant ANR‐21‐CE16‐0003‐01 PARK‐PEP, Fondation de France (Maladie de Parkinson, R19199EK), France Parkinson (R16008), and The Michael J. Fox Foundation, grant numbers 6709.03, 10255.03, and 12938.04, and the Protocole Hospitalier de Recherches Cliniques Convergence (CPP/2008/009).

## Author Roles

(1) Research Project: A. Conception, B. Organization, C. Execution, D. Data Curation; (2) Statistical Analysis: A. Design, B. Execution, C. Review and Critique; (3) Manuscript Preparation: A. Writing of the First Draft, B. Review and Critique

I.C.: 1A, 1C, 1D, 2B, 3A

E.M.: 1B, 1C, 1D, 3B

S.E.: 1B, 1D

S.C.: 1B, 1D

A.M.: 1B, 1D

C.D.: 1B, 1D

L.G.: 1B, 1D

G.B.: 1B, 1D

M.T.: 1B, 3B

R.M.: 1C, 1D, 3A

X.T.: 1B, 3B

G.V.: 1D, 3B

W.V.: 1D, 3B

L.B.: 1B, 3B

L.D.: 1B, 3B

B.S.: 1B, 3B

M.‐C.C.‐H.: 1A, 1B, 2B, 3B

J.‐M.T.: 1A, 2A, 2B, 3B

V.H.: 1A, 1B, 1D, 2A, 2B, 3A, 3B

## Financial Disclosures of All Authors

I.C.: None.

E.M.: Protocole Hospitalier de Recherches Cliniques Convergence (CPP/2008/009).

S.E.: None.

S.C.: None.

A.M.: None.

C.D.: None.

L.G.: None.

G.B.: None.

M.T.: None.

R.M.: None.

X.T.: The CANTHER laboratory “CANcer, heterogeneity, plasticity and resistance to THERapies” is supported by a grant from “Contrat de Plan Etat‐Région” Cancer 2015–2020.

G.V.: None.

W.V.: FWO (Research Foundation – Flanders), grants G0D5217N and G072821N.

L.B.: Programs d'Investissements d'Avenir LabEx (excellence laboratory) DISTALZ (Development of Innovative Strategies for a Transdisciplinary approach to ALZheimer's disease).

L.D.: Board: Abbvie; Honoraires symposium: UCB, Abbvie, Orkyn.

B.S.: None.

M.‐C.C.‐H.: ANR (Agence Nationale de Recherche, France) grant ANR‐16‐CE16‐0012‐02 MeTDePaDi, grant ANR‐20‐CE16‐0008 Synapark, grant ANR‐21‐CE16‐0003‐01 PARK‐PEP, Fondation de France (Maladie de Parkinson, R19199EK), France Parkinson (R16008), Association des Aidants et Malades à Corps de Lewy (A2MCL) charity, France Alzheimer charity, and The Michael J. Fox Foundation, grant numbers 6709.03, 10255.03, and 12938.04, and the Protocole Hospitalier de Recherches Cliniques Convergence (CPP/2008/009).

J.‐M.T.: ANR (Agence Nationale de Recherche, France) grant ANR‐16‐CE16‐0012‐02 MeTDePaDi, grant ANR‐20‐CE16‐0008 Synapark, grant ANR‐21‐CE16‐0003‐01 PARK‐PEP, Fondation de France (Maladie de Parkinson, R19199EK), France Parkinson (R16008), and The Michael J. Fox Foundation, grant numbers 6709.03, 10255.03, and 12938.04.

V.H.: Association des Aidants et Malades à Corps de Lewy (A2MCL) charity, France Alzheimer charity, Programs d'Investissements d'Avenir LabEx (excellence laboratory) DISTALZ (Development of Innovative Strategies for a Transdisciplinary approach to ALZheimer's disease).

## Supporting information


**Appendix S1** Supporting Information

**TABLE S1** List of primer names and sequences used for site‐directed‐mutagenesis

**TABLE S2** Antibodies used in immunohistochemistry experiments

**TABLE S3** Results of the mutation prediction tools

FIG. S1

FIG. S2

FIG. S3

FIG. S4

## Data Availability

The data that support the findings of this study are available from the corresponding authors upon reasonable request.
