# Supplementary material for: Functional Analyses of Two Novel LRRK2 Pathogenic Variants in Familial Parkinson′s Disease
Source: Mov Disord. 2022 Jun 16;37(8):1761–7. doi: 10.1002/mds.29124 (PMC9543145; doi:10.1002/mds.29124)
Supplement: Supplementary file 2 — TABLE S1 List of primer names and sequences used for site‐directed‐mutagenesis [file MDS-37-1761-s005.docx]

## **Supplementary Table S1. List of primer names and sequences used for site-directed-mutagenesis.**

| **Primer name** | **Sequence** |
| --- | --- |
| LRRK2-H230R-F | GGAATCGCTAGGGAACGTAAACAATGCAGCACATGA |
| LRRK2-H230R-R | TCATGTGCTGCATTGTTTACGTTCCCTAGCGATTCC |
| LRRK2-A1440P-F | ACAGGGGAAGAAGAAGCGCGAGGCTTTATATTGAAGAGCC |
| LRRK2-A1440P-R | GGCTCTTCAATATAAAGCCTGGCGCTTCTTCTTCCCCTGT |
| LRRK2-G2019S-F | GCAGTACTGAGCAATGCTGTAGTCAGCAATCTTTG |
| LRRK2-G2019S-R | CAAAGATTGCTGACTACAGCATTGCTCAGTACTGC |
| LRRK2-R1441G-F | GAAGAAGAAGCGCCAGCCTTTATATTGAAGAGCCAAG |
| LRRK2- R1441G-R | CTTGGCTCTTCAATATAAAGGCTGGCGCTTCTTCTTC |
