# Supplementary material for: Functional Analyses of Two Novel LRRK2 Pathogenic Variants in Familial Parkinson′s Disease
Source: Mov Disord. 2022 Jun 16;37(8):1761–7. doi: 10.1002/mds.29124 (PMC9543145; doi:10.1002/mds.29124)
Supplement: Supplementary file 3 — TABLE S2 Antibodies used in immunohistochemistry experiments [file MDS-37-1761-s004.docx]

## **Supplementary table S2. Antibodies used in immunohistochemistry experiments**

H+L = Heavy chain + Light chain.

| **Antibody** | **Species** | **Dilution** | **Secondary** | **Source** |
| --- | --- | --- | --- | --- |
| LRRK2 | Rabbit | 1/1 000 | Goat anti-rabbit IgG Antibody (H+L), Peroxidase | Abcam (ab133518)  (Abcam, Cambridge, UK) |
| Flag M2 | Mouse | 1/1 000 | Horse anti-mouse IgG Antibody (H+L), Peroxidase | Sigma (A2220)  (Sigma-Aldrich, Saint-Louis, MI, USA) |
| pSer935 LRRK2 | Rabbit | 1/1 000 | Goat anti-rabbit IgG Antibody (H+L), Peroxidase | Abcam (ab133450)  (Abcam, Cambridge, UK) |
| pSer910 LRRK2 | Rabbit | 1/1 000 | Goat anti-rabbit IgG Antibody (H+L), Peroxidase | Abcam (ab133449)  (Abcam, Cambridge, UK) |
| pSer1292 LRRK2 | Rabbit | 1/500 | Goat anti-rabbit IgG Antibody (H+L), Peroxidase | Abcam (ab203181)  (Abcam, Cambridge, UK) |
| RAB10 | Rabbit | 1/1 000 | Goat anti-rabbit IgG Antibody (H+L), Peroxidase | Cell signaling (D36C4)  (Cell Signaling, Danvers, MA, USA) |
| pThr73 RAB10 | Rabbit | 1/500 | Goat anti-rabbit IgG Antibody (H+L), Peroxidase | Abcam (ab230260)  (Abcam, Cambridge, UK) |
| GAPDH | Rabbit | 1/50 000 | Goat anti-rabbit IgG Antibody (H+L), Peroxidase | Sigma (G9545)  (Sigma-Aldrich, Saint-Louis, MI, USA) |
| anti-mouse IgG antibody (H+L) | Horse | 1/50 000 | - | Vector Laboratories (PI-2000)  (Vector Laboratories, Burlingame, CA, USA) |
| anti-rabbit IgG Antibody (H+L), Peroxidase | Goat | 1/50 000 | - | Vector Laboratories (PI-1000)  (Vector Laboratories, Burlingame, CA, USA) |
