# Supplementary material for: Functional Analyses of Two Novel LRRK2 Pathogenic Variants in Familial Parkinson′s Disease
Source: Mov Disord. 2022 Jun 16;37(8):1761–7. doi: 10.1002/mds.29124 (PMC9543145; doi:10.1002/mds.29124)
Supplement: Supplementary file 4 — TABLE S3 Results of the mutation prediction tools [file MDS-37-1761-s003.docx]

## **Supplementary Table S3. Results of the mutation prediction tools**

| Variant | DANN_score^1^ | FATHMM_pred | GERP++_RS^2^ | LRT  _pred | MetaLR  _pred | MetaSVM  _pred | MutationTaster  _pred | PROVEAN  _pred | SIFT | PolyPhen2 | MutationAssessor  _pred | REVEL score^3^ |
| --- | --- | --- | --- | --- | --- | --- | --- | --- | --- | --- | --- | --- |
| H230R | **0.987** | Tolerated | **5.65** | Neutral | Tolerated | Tolerated | Polymorphism | Neutral | Tolerated | **Possibly damaging** | Medium | 0.124 |
| A1440P | **0.998** | Tolerated | **5.64** | **Deleterious** | **Damaging** | **Damaging** | **Disease causing** | **Damaging** | **Deleterious** | **Probably damaging** | Medium | **0.702** |

^1^ DANN scores range from 0 to 1 and a larger number indicate a higher probability to be damaging.

^2^ The larger the GERP++ RS score, the more conserved the site. Scores range from -12.3 to 6.17.

^3^ The larger the REVEL score, the more the variant is predicted to be pathogenic. Scores range from 0 to 1.
