# Supplementary figures and images for: Functional Analyses of Two Novel LRRK2 Pathogenic Variants in Familial Parkinson′s Disease
Source: Mov Disord. 2022 Jun 16;37(8):1761–7. doi: 10.1002/mds.29124 (PMC9543145; doi:10.1002/mds.29124)

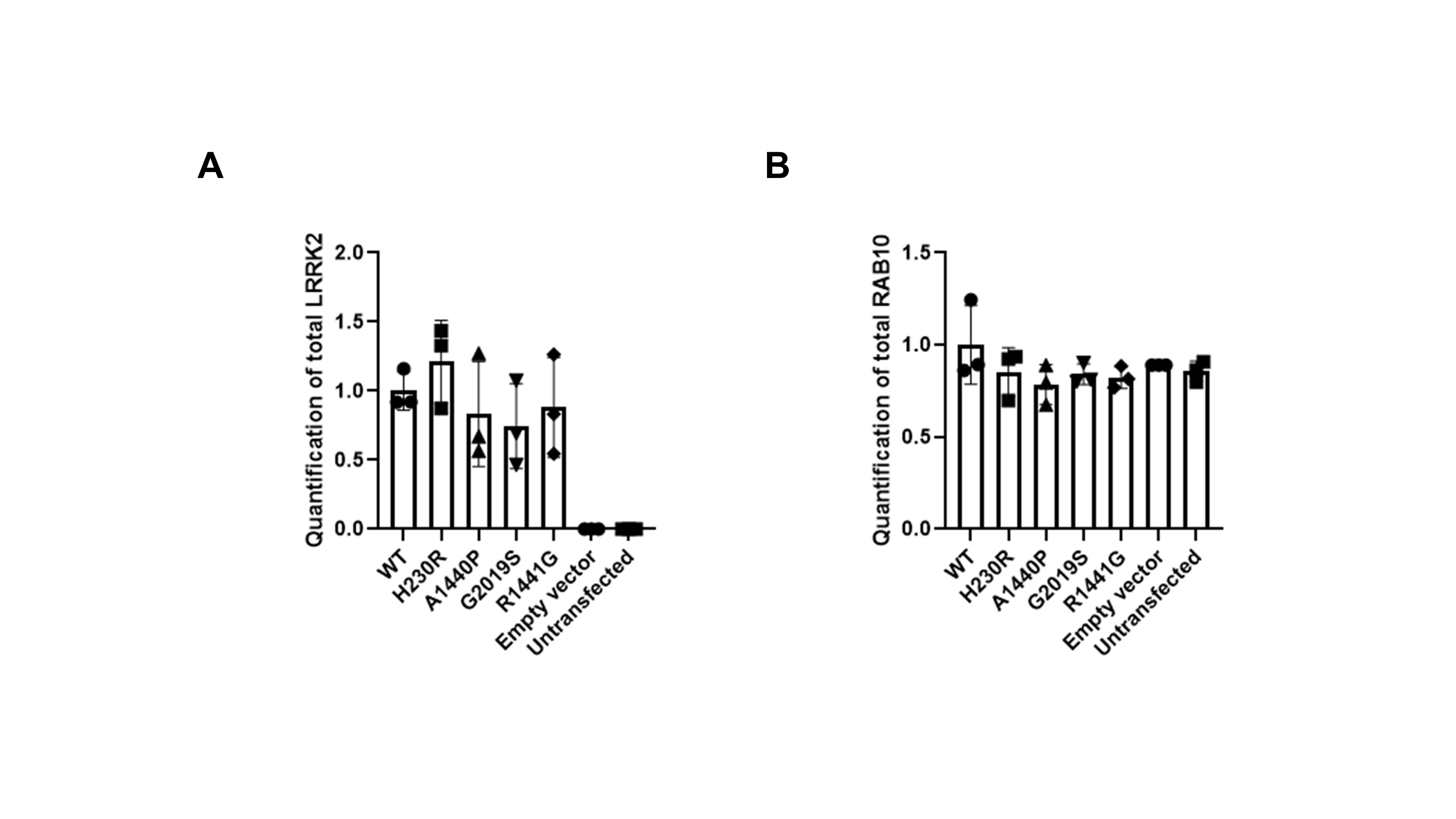

Supplement: Supplementary file 5 — FIG. S1 [file MDS-37-1761-s001.tif]

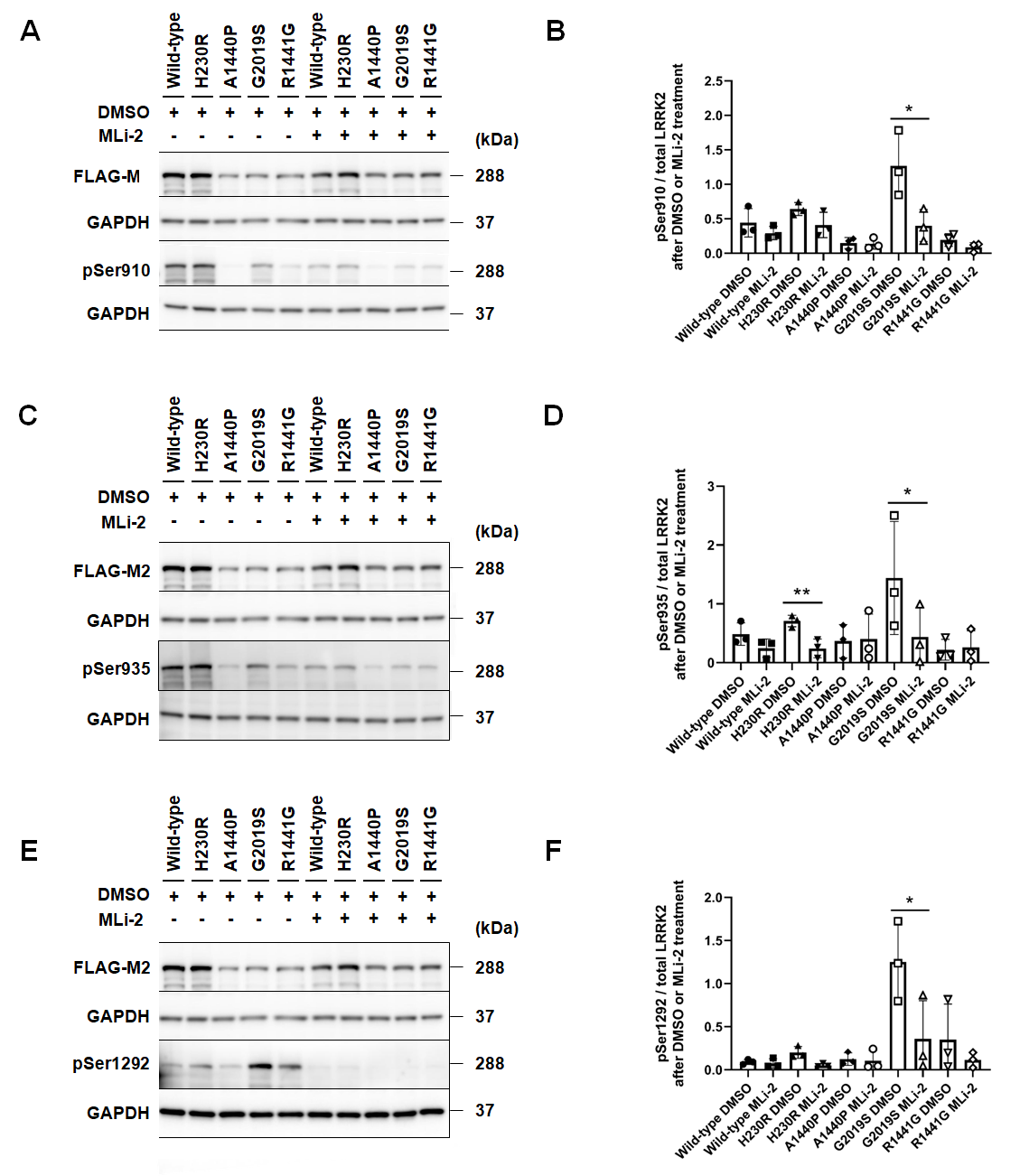

Supplement: Supplementary file 6 — FIG. S2 [file MDS-37-1761-s002.tif]

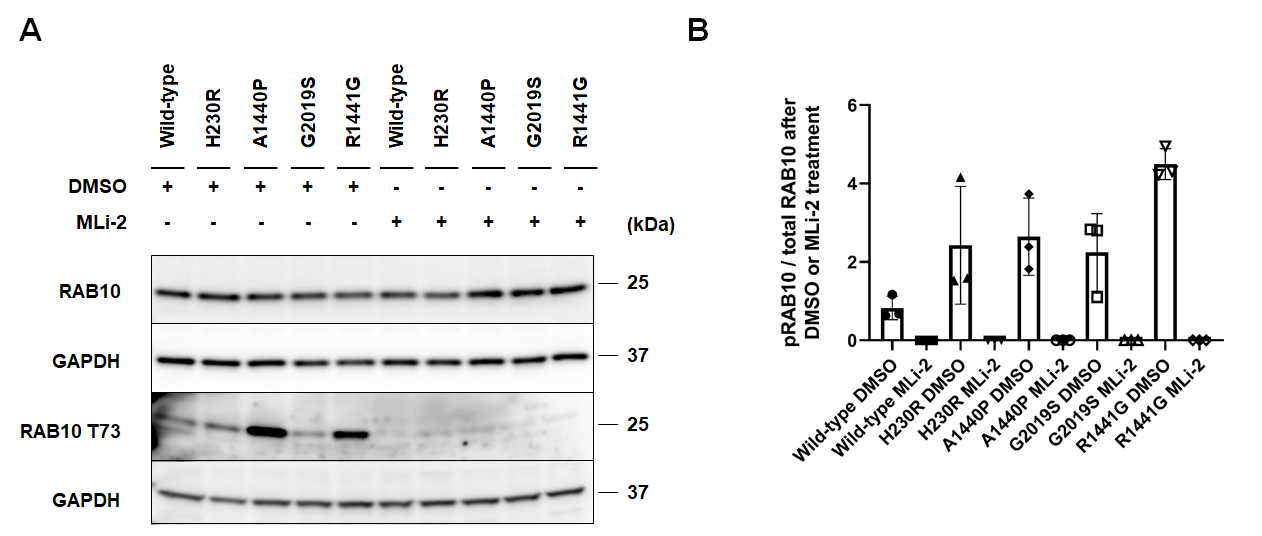

Supplement: Supplementary file 7 — FIG. S3 [file MDS-37-1761-s006.tif]

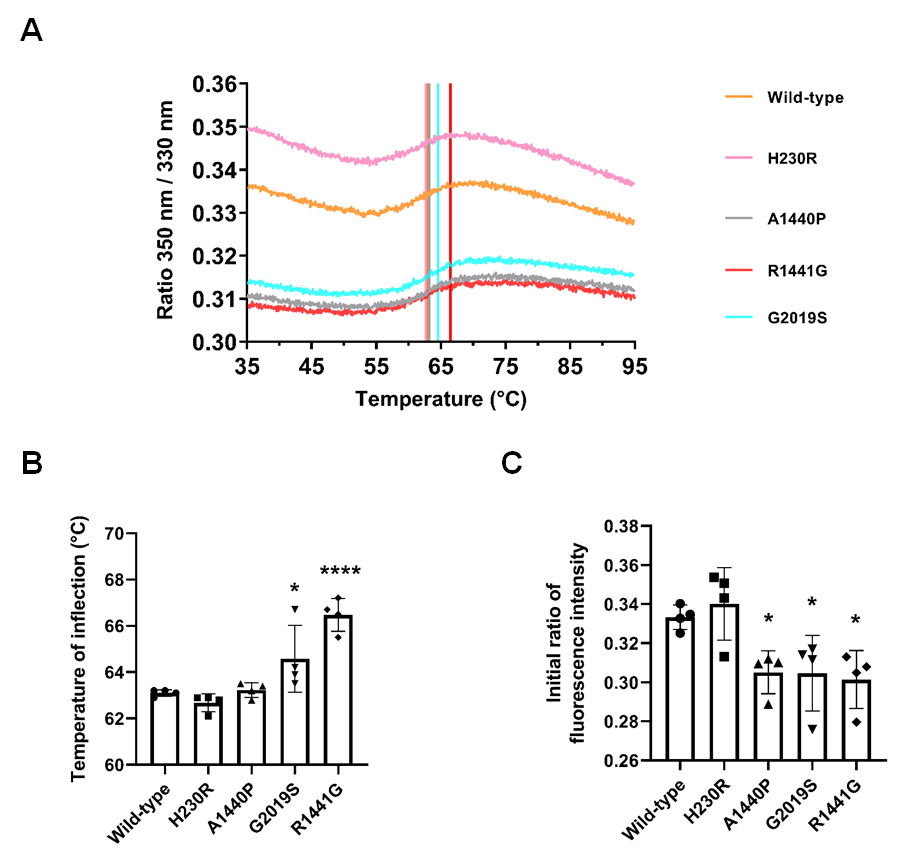

Supplement: Supplementary file 8 — FIG. S4 [file MDS-37-1761-s007.tif]
